# Clinical Implication of Supra-Normal Left Ventricular Ejection Fraction in Patients Undergoing Transcatheter Aortic Valve Replacement

**DOI:** 10.3390/jcm12237429

**Published:** 2023-11-30

**Authors:** Teruhiko Imamura, Yuki Hida, Hiroshi Ueno, Koichiro Kinugawa, Fumiaki Yashima, Norio Tada, Masahiro Yamawaki, Shinichi Shirai, Toru Naganuma, Futoshi Yamanaka, Masahiko Noguchi, Kazuki Mizutani, Kensuke Takagi, Yusuke Watanabe, Masanori Yamamoto, Masahiko Asami, Masaki Izumo, Yohei Ohno, Hidetaka Nishida, Kentaro Hayashida, on behalf of the OCEAN-TAVI Investigators

**Affiliations:** 1 Second Department of Internal Medicine, University of Toyama, Toyama 930-0194, Japanhueno@med.u-toyama.ac.jp (H.U.);; 2 Department of Cardiology, Saiseikai Utsunomiya Hospital, Utsunomiya 321-0974, Japan; 3 Department of Cardiology, Sendai Kosei Hospital, Sendai 980-0873, Japan; 4 Department of Cardiology, Saiseikai Yokohama City Eastern Hospital, Yokohama 230-0012, Japan; 5 Department of Cardiovascular Medicine, Kokura Memorial Hospital, Kitakyushu 802-8555, Japan; 6 Department of Cardiology, New Tokyo Hospital, Matsudo 270-2232, Japan; 7 Department of Cardiology, Shonan Kamakura General Hospital, Kamakura 247-8533, Japan; 8 Department of Cardiology, Tokyo Bay Urayasu Ichikawa Medical Center, Urayasu 279-0001, Japan; m.noguchi.0918@gmail.com; 9 Department of Cardiology, Faculty of Medicine, Kindai University, Osaka 589-8511, Japan; 10 Department of Cardiovascular Medicine, National Cerebral and Cardiovascular Center, Osaka 564-8565, Japan; 11 Department of Cardiology, Teikyo University School of Medicine, Tokyo 173-8606, Japan; 12 Department of Cardiology, Toyohashi Heart Center, Toyohashi 441-8071, Japan; 13 Department of Cardiology, Nagoya Heart Center, Nagoya 461-0045, Japan; 14 Division of Cardiology, Mitsui Memorial Hospital, Tokyo 101-8643, Japan; 15 Department of Cardiology, St. Marianna University School of Medicine, Kawasaki 216-8511, Japan; 16 Department of Cardiology, Tokai University School of Medicine, Isehara 259-1193, Japan; 17 Department of Cardiology, Tsukuba Medical Center Hospital, Tsukuba 305-8558, Japan; 18 Department of Cardiology, Keio University School of Medicine, Tokyo 160-8582, Japan

**Keywords:** aortic valve disease, heart failure, endovascular intervention

## Abstract

Background: Individuals with heart failure displaying supra-normal left ventricular ejection fraction (snLVEF) may exhibit less favorable clinical outcomes in contrast to their counterparts with normal left ventricular ejection fraction (nLVEF). The distinctive characteristics and mid-term prognosis of individuals with severe aortic stenosis and snLVEF following transcatheter aortic valve replacement (TAVR) remain enigmatic. Methods: Among 7393 patients diagnosed with severe aortic stenosis who underwent TAVR between 2013 and 2019 and were enlisted in the optimized transcatheter valvular intervention (OCEAN-TAVI) multicenter registry (UMIN000020423), we selected patients with left ventricular ejection fraction (LVEF) ≥ 50%. snLVEF was defined as LVEF exceeding 65%. We compared the baseline characteristics and assessed three-year post-TAVR mortality and heart failure readmission rates between the snLVEF (LVEF > 65%) and nLVEF cohorts (LVER 50–65%). Results: Our study cohort comprised 5989 patients (mean age 84.4 ± 5.1 years and 1783 males). Among these, 2819 patients were categorized within the snLVEF cohort, while the remaining 3170 were allocated to the nLVEF group. Individuals within the snLVEF cohort were more likely to be female and displayed lower levels of natriuretic peptides, as well as smaller left ventricular dimensions in comparison to their nLVEF counterparts (*p* < 0.05 for all). The presence of snLVEF emerged as an independent predictor of the three-year composite endpoint relative to nLVEF, with an adjusted hazard ratio of 1.16 (95% confidence interval 1.02–1.31, *p* = 0.023) after accounting for several potential confounding factors. Conclusions: snLVEF was relatively common among candidates for TAVR with preserved ejection fraction. Patients harboring snLVEF appear to manifest a distinctive clinical profile and encounter less favorable clinical outcomes following TAVR in contrast to those characterized by nLVEF.

## 1. Introduction

Recent guidelines have undergone revisions in the sub-group categorization of left ventricular ejection fraction (LVEF), which now distinguishes heart failure (HF) into distinct categories: HF with reduced LVEF (HFrEF), HF with mildly reduced LVEF (HFmrEF), and HF with preserved LVEF (HFpEF) [1,2,3]. The diagnosis of HF hinges upon the manifestation of clinical symptoms and signs, with therapeutic strategies tailored to the classification of LVEF [4]. 

Numerous medications designed to ameliorate HF have demonstrated their efficacy in enhancing both mortality and morbidity outcomes among patients with HFrEF and HFmrEF [1,2,3]. However, the therapeutic benefits of these agents tend to diminish with increasing LVEF among individuals with HfpEF [5,6,7]. Notably, recent investigations have revealed that patients with LVEF values exceeding the 60–65% range exhibit a higher mortality rate compared to those with a normal LVEF (nLVEF) [8,9,10]. In light of these findings, the concept of “supra-normal LVEF (snLVEF)”, conventionally defined as an LVEF exceeding 65%, has been introduced [11]. 

Patients with severe aortic stenosis often experience HF symptoms attributable to heightened afterload imposed on the left ventricles, even though most of them retain a preserved LVEF [12]. These symptoms may persist even following trans-catheter aortic valve replacement (TAVR) due to refractory extra-valvular impairments and are associated with mortality and morbidity [13]. Importantly, a subset of TAVR candidates may exhibit snLVEF. Nevertheless, there is no previous literature that investigated the profiles and prognostic impact of snLVEF in TAVR candidates. Our hypothesis posits that patients with snLVEF may possess a unique clinical profile and divergent clinical outcomes after TAVR relative to their nLVEF counterparts. This knowledge holds the potential to augment our risk stratification capabilities and facilitate more nuanced shared decision-making processes prior to TAVR. 

In this pioneering study, we embarked on an investigation utilizing large-scale multi-center registry data, thereby delving into the clinical characteristics and post-TAVR clinical trajectories of individuals distinguished by snLVEF (LVEF > 65%) and nLVEF (LVEF 50–65%). 

## 2. Methods

### 2.1. Patient Selection

A total of 7393 patients presenting with severe aortic stenosis, defined by criteria as encompassing an aortic valve area < 1.0 cm^2^, mean pressure gradient exceeding 40 mmHg, and/or peak velocity surpassing 4.0 m/s, either at rest or during dobutamine loading, underwent TAVR at multiple high-volume medical centers in Japan between 2013 and 2019. These patients were meticulously enrolled in the prospective optimized catheter valvular intervention–transcatheter aortic valve implantation (OCEAN-TAVI) multicenter registry (UMIN000020423). 

Among this cohort, individuals exhibiting a baseline LVEF below 50% were systematically excluded, and our retrospective study exclusively encompassed those with an LVEF equal to or exceeding 50% at baseline. The research protocol garnered the requisite approval from the local ethics committees at each participating institution, with written informed consent being diligently obtained from all participants before their enrollment in the registry. 

### 2.2. Study Design

Patients were categorized into two cohorts based on their baseline LVEF levels before TAVR using a cutoff value of 65%: snLVEF group, comprising individuals with LVEF > 65%, and nLVEF group, encompassing those with LVEF values ranging from 50% to 65%. All patients were followed for three years after TAVR. A primary outcome was a composite of all-cause death and heart failure readmission. Comprehensive comparisons were made between the two cohorts concerning patients’ clinical profiles and subsequent clinical outcomes. 

### 2.3. TAVR Procedure

The eligibility for TAVR was established by the multidisciplinary heart valve team at each participating institution. Patients underwent the TAVR procedure using Sapien 3 (Edwards Life Sciences Inc., Irvine, CA, USA), Evolut R (Medtronic, Dublin, Ireland), Sapien XT (Edwards Life Sciences Inc.), or CoreValve (Medtronic) via various approaches, including trans-femoral, trans-apical, transiliac, trans-subclavian, or direct aortic routes, and the procedure was performed under either general or local anesthesia. 

### 2.4. Variables Evaluated

Demographic information, comorbidities, laboratory results, and echocardiographic data obtained before TAVR procedure were compiled as baseline characteristics. The presence of snLVEF before TAVR was designated as the independent variable. 

Data regarding peri-procedural and in-hospital complications were systematically documented. Subsequently, patients were subjected to a three-year follow-up period after TAVR, either at outpatient clinics affiliated with the participating institutions or at their associated healthcare centers, under scheduled appointments or as needed. The primary composite outcome encompassed all-cause mortality and HF readmission. Efforts were made to ascertain the causes of death in each case. 

### 2.5. Statistical Analysis

A significance level of *p* < 0.05 was employed to establish statistical significance. All statistical analyses were conducted utilizing SPSS Statistics 23 (SPSS Inc., Armonk, IL, USA). Continuous variables, following confirmation of their normal distribution, were expressed as means and standard deviations, and between-group comparisons were executed with unpaired *t*-tests. Categorical variables were presented as counts and percentages, with comparisons conducted using either the Chi-square test or Fisher’s exact test as deemed appropriate. 

The independent variable was characterized as snLVEF, denoting baseline LVEF exceeding 65%. The dependent variable was defined as a composite outcome encompassing all-cause mortality and HF readmission during a 3-year observation period after TAVR. 

Receiver operating characteristic analysis was conducted to assess the predictive capability of baseline LVEF for the primary composite outcome. The cumulative incidence of clinical outcomes in the two cohorts (snLVEF group versus nLVEF group) was compared using the log-rank test. Furthermore, Cox proportional hazard ratio regression analyses were performed to evaluate the prognostic impact of snLVEF on clinical outcomes. These analyses were adjusted for predetermined potential confounding factors, including age, male sex, the presence of chronic kidney disease, and the logarithm of plasma B-type natriuretic peptide levels.

## 3. Results

### 3.1. Baseline Characteristics

**Figure 1:**
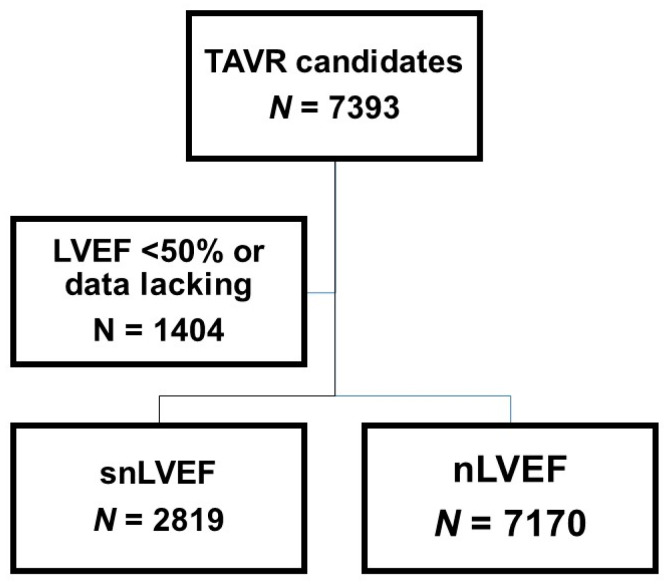
Flow chart of this study. A total of 5989 patients with LVEF ≥ 50% who underwent TAVR were finally included by excluding those with LVEF < 50% and those with data lacking. Patients were stratified into two groups according to their LVEF: snLVEF with LVEF > 65% and nLVEF with LVEF 50–65%. LVEF, left ventricular ejection fraction; TAVR, transcatheter aortic valve replacement; snLVEF, supra-normal left ventricular ejection fraction; nLVEF, normal left ventricular ejection fraction.

A total of 7393 patients were initially identified for inclusion in the OCEAN registry database. Subsequently, 1404 patients with LVEF below 50% or those lacking LVEF data were excluded from the study (Figure 1). Consequently, the final cohort comprised 5989 patients with LVEF values equal to or exceeding 50% (Table 1). The mean age of this cohort was 84.4 ± 5.1 years, with 1783 individuals (30%) being of male gender. Notably, approximately 40% of these patients exhibited New York Heart Association class III/IV symptoms. A substantial proportion of the cohort presented with multiple comorbidities, including atrial fibrillation (1202 patients [20%]), chronic kidney disease (4078 patients [68%]), and chronic obstructive pulmonary disease (569 patients [10%]). The mean logarithmic value of plasma B-type natriuretic peptide was mildly elevated at 2.28 ± 0.46 pg/mL. Per this study’s inclusion criteria, all patients exhibited an LVEF equal to or exceeding 50%, with a mean LVEF of 64.8 ± 7.3%. Additionally, approximately 10% of the patients presented with concomitant valvular diseases of moderate or greater severity, respectively. 

### 3.2. Baseline LVEF Distribution

**Figure 2:**
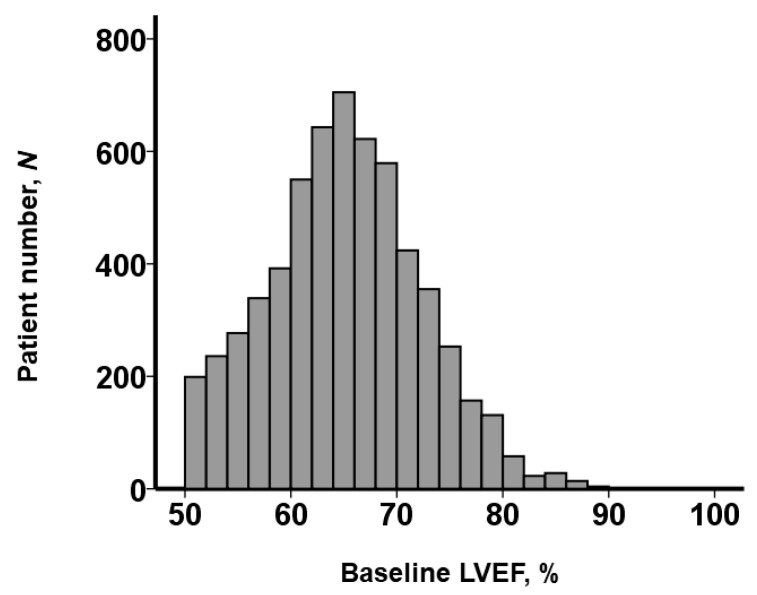
Distribution of baseline LVEF obtained before TAVR. Per the study protocol, all patients had LVEF ≥ 50%. Of them, 2819 patients had snLVEF, and 3170 patients had nLVEF. LVEF, left ventricular ejection fraction; TAVR, trans-catheter aortic valve replacement; snLVEF, supra-normal left ventricular ejection fraction; nLVEF, normal left ventricular ejection fraction. snLVEF was defined as LVEF > 65%. nLVEF was defined as LVEF 50–65%.

Baseline LVEF exhibited a wide distribution spanning from 50% to 90%, as illustrated in Figure 2. Among these patients, 2819 individuals (47%) possessed LVEF values exceeding 65% and were thus categorized into the snLVEF group. Accordingly, patients were segregated into two distinct cohorts based on their baseline LVEF: the snLVEF group, comprised of those with LVEF > 65% (*N* = 2819), and the nLVEF group, consisting of individuals with LVEF ≤ 65% (*N* = 3170). 

The snLVEF group exhibited a distinctive clinical profile in comparison to the nLVEF group, as outlined in Table 1. Individuals within the snLVEF cohort were more frequently female, presented with milder symptoms, showcased lower levels of plasma B-type natriuretic peptide, displayed a decreased prevalence of ischemic heart disease history, and possessed smaller left ventricular dimensions relative to their nLVEF counterparts (*p* < 0.05 for all). 

### 3.3. Peri-Procedural Complication

A limited number of patients experienced peri-procedural complications, as summarized in Table 2. Notably, the rates of complications did not significantly differ between the two cohorts, except for instances of mitral valve injury (*p* = 0.029). Furthermore, in-hospital complication rates remained largely comparable between the two groups, except for the incidence of new-onset atrial fibrillation, where a statistically significant difference was observed (*p* = 0.029). 

### 3.4. One-Year Follow-Up of Echocardiography

Comprehensive echocardiographic data at 1-year follow-up were obtained from 657 patients, consisting of 405 patients with snLVEF and 252 patients with nLVEF (Table 3). Left ventricular outflow tract (LVOT) peak velocity was significantly higher in the snLVEF group (1.07 ± 0.32 versus 0.98 ± 0.23 m/s, *p* = 0.017). There were nine patients who had LVOT peak velocity > 2.0 m/s, all of whom were assigned to the snLVEF group (*p* = 0.017). 

### 3.5. Mid-Term Clinical Outcome

**Figure 3:**
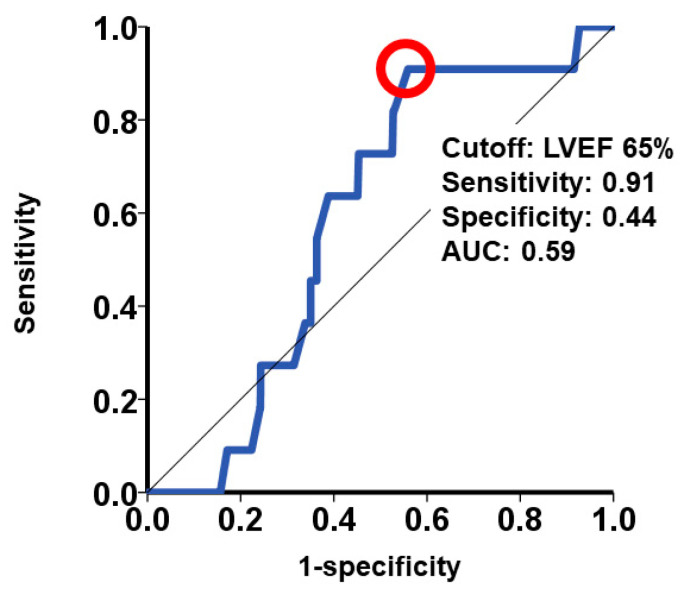
Receiver operating characteristics analysis for baseline LVEF to estimate the 3-year composite outcome consisting of death and heart failure readmission. A cutoff of baseline LVEF was calculated as 65% and pointed as a red circle. LVEF, left ventricular ejection fraction.

**Figure 4:**
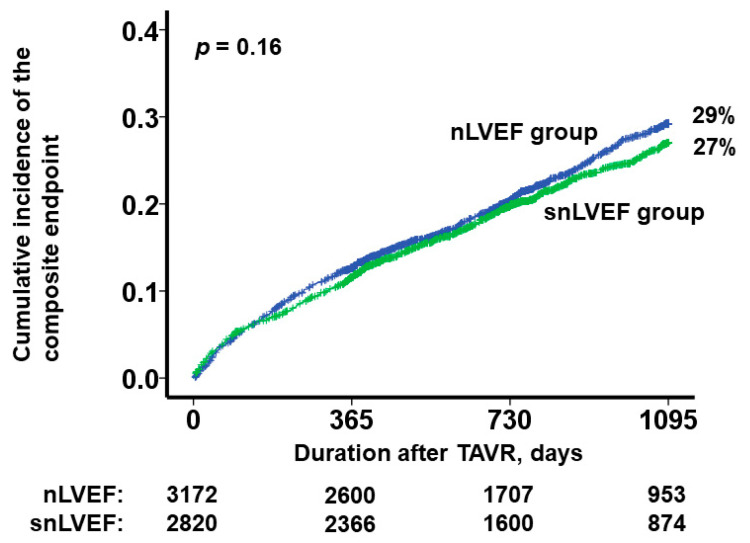
Three-year cumulative incidence of the composite endpoint (death and heart failure readmission) after TAVR, which was stratified by the presence of snLVEF. TAVR, trans-catheter aortic valve replacement; snLVEF, supra-normal left ventricular ejection fraction; nLVEF, normal left ventricular ejection fraction. snLVEF was defined as LVEF > 65%. nLVEF was defined as LVEF 50–65%. Two curves were compared using log-rank test.

A threshold baseline LVEF of 65% was determined to predict the primary outcome, yielding a sensitivity of 0.91, a specificity of 0.44, and an area under the curve of 0.59 (Figure 3). Notably, the cumulative incidence of the primary outcome did not exhibit a statistically significant difference between the two groups (*p* = 0.16; Figure 4). Similarly, the cumulative incidence of both all-cause mortality and HF readmission demonstrated no significant distinctions between the two cohorts (*p* = 0.16 and *p* = 0.12, respectively). Among the total of 1010 recorded deaths, 127 out of 458 were attributed to cardiovascular causes in the snLVEF group, while 154 out of 552 were attributed to cardiovascular causes in the nLVEF group (*p* = 0.51). 

Initially, snLVEF did not exhibit a statistically significant association with the primary outcome, as indicated by an unadjusted hazard ratio of 0.93 (95% confidence interval 0.83–1.03, *p* = 0.16). However, after adjusting for potential confounding factors (as outlined in Table 4), snLVEF achieved statistical significance with an adjusted hazard ratio of 1.16 (95% confidence interval 1.02–1.31, *p* = 0.023), together with all other included potential confounders. This prognostic impact of snLVEF was found to be significant in the context of mortality but not concerning HF readmission (*p* = 0.014 and *p* = 0.64, respectively). 

**Figure 5:**
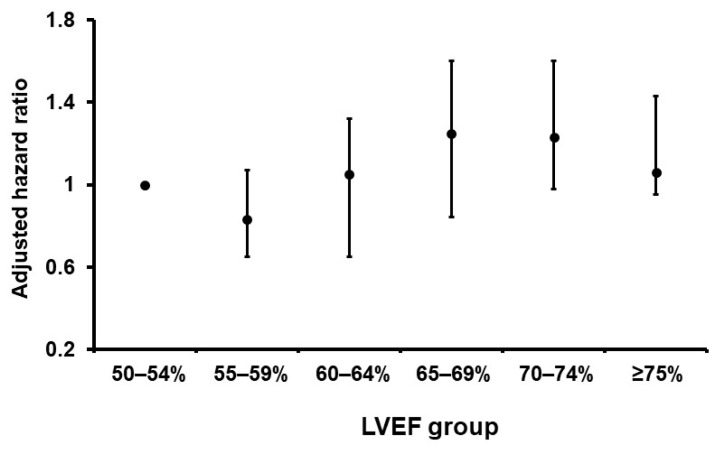
Adjusted hazard ratio in each LVEF group per 5% for the 3-year composite endpoint. Patients with LVEF 50–54% were defined as a reference. Patients with LVEF 55–59% had a nadir and those with LVEF 65–69% had the highest risk. Hazard ratios were adjusted for age, male sex, the presence of chronic kidney disease, and the logarithm of plasma B-type natriuretic peptide level. LVEF, left ventricular ejection fraction.

The adjusted hazard ratio for each LVEF group, stratified at intervals of 5%, is presented in Figure 5. LVEF falling within the range of 50% to 54% served as the reference point. Notably, the LVEF group spanning 55% to 59% exhibited the lowest risk, while the cohort with LVEF values between 65% and 69% displayed the highest risk.

### 3.6. Further Risk Stratification

**Figure 6:**
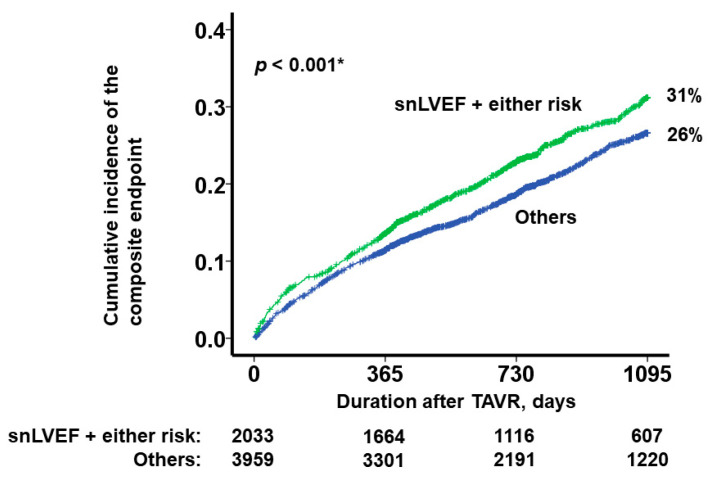
Three-year cumulative incidence of the composite endpoint (death and heart failure readmission) after TAVR, which was stratified by the presence of both snLVEF and at least one risk factor. TAVR, trans-catheter aortic valve replacement; snLVEF, supra-normal left ventricular ejection fraction. snLVEF was defined as LVEF > 65%. nLVEF was defined as LVEF 50–65%. Two curves were compared using log-rank test. We pre-specified four risk factors: age > 85 years, male sex, the logarithm of B-type natriuretic peptide exceeding 2.0, and estimated glomerular filtration rate <30 mL/min/1.73 m^2^. Patients who exhibited both snLVEF and at least one risk factor were assigned to the high-risk group. * *p* <0.05.

For additional risk stratification, we employed snLVEF in conjunction with predetermined risk factors that were used in the multivariable analysis, including advanced age (>85 years), male gender, a logarithmically transformed B-type natriuretic peptide level exceeding 2.0, and an estimated glomerular filtration rate below 30 mL/min/1.73 m^2^. Patients who exhibited both snLVEF and at least one of these risk factors were categorized into the high-risk cohort (*N* = 2033). This group demonstrated a significantly elevated cumulative incidence of the primary endpoint compared to their counterparts (31% versus 26%, *p* < 0.001; Figure 6).

## 4. Discussion

In this retrospective analysis utilizing prospectively collected data from the extensive multi-center OCEAN-TAVI registry database, we embarked on an investigation into the clinical profile and subsequent clinical outcomes of patients with severe aortic stenosis who presented with a baseline snLVEF, defined as LVEF exceeding 65%. Our primary objective was to compare their profiles and outcomes with those of patients possessing baseline nLVEF, designated as LVEF ranging from 50% to 65%, over a three-year observational period following TAVR.

Our study unveiled that snLVEF was a relatively common occurrence among TAVR candidates characterized by preserved ejection fraction. Patients within the snLVEF group were more likely to be female, exhibited a lower frequency of ischemic heart disease, displayed reduced levels of natriuretic peptides, and manifested smaller left ventricular dimensions in contrast to their nLVEF counterparts. Importantly, the presence of baseline snLVEF emerged as an independent predictor of a combined endpoint encompassing three-year mortality and HF readmission rates after TAVR. The presence of snLVEF was associated with a higher risk, particularly in patients with either of conventional risk factors such as old age. 

### 4.1. The Concept and Cutoff of snLVEF

The notion of snLVEF has only recently emerged, initially arising from the analysis of a substantial dataset derived from routine clinical echocardiography practice [8]. This comprehensive study revealed that individuals with LVEF values falling within the 60–65% range exhibited the lowest mortality rates. Intriguingly, this trend persisted even after excluding individuals with acute illnesses that may potentially increase LVEF, such as sepsis and hypovolemia, and following adjustments for various potential confounding factors that may contribute to elevated LVEF, such as mitral regurgitation, hypertrophy, anemia, and hyperthyroidism. Additional studies have also lent support to the concept of snLVEF in cohorts with HF [9,10]. 

It is essential to recognize that the choice of a specific cutoff value for defining snLVEF is somewhat arbitrary. In our study, we adopted the cutoff of LVEF at 65% based on prior literature that observed an increased mortality risk among individuals with LVEF values exceeding this threshold [8]. Additionally, the treatment effects of several HF medications have demonstrated consistency across the entire spectrum of LVEF but have shown attenuation in patients with LVEF values exceeding 65% [5,6,7]. Remarkably, in line with previous findings, an LVEF of 65% was identified as a statistically significant cutoff for predicting death or HF readmission in our cohort.

### 4.2. The Unique Profile of the TAVR Candidates with snLVEF

Interestingly, several studies have also identified similar distinctive profiles in HF patients exhibiting snLVEF [9,10]. These individuals tend to be older, predominantly female, possess lower levels of natriuretic peptides, exhibit a higher prevalence of non-ischemic etiologies, and present with smaller left ventricular dimensions in comparison to their counterparts with nLVEF. 

Our cohort with snLVEF had almost consistent profiles, except for age, probably because most of the TAVR candidates were elderly patients, as expected [14]. It is not surprising because many TAVR candidates may have HFpEF after the improvement of the stenotic aortic valve via TAVR due to persistent extra-valvular impairment such as concentric hypertrophy [13]. 

These variables are famous as unique profiles of HFpEF, consisting of HF with nLVEF and HF with snLVEF [15]. Thus, patients with snLVEF may be more specific representative cohorts of conventional HFpEF. 

### 4.3. Prognostic Impact of snLVEF after TAVR

In line with previous research conducted in HF cohorts [9,10], our study has established that the presence of snLVEF independently correlates with mortality and morbidity following TAVR. This finding underscores the clinical relevance of snLVEF in predicting patient outcomes post-TAVR. Clinically, snLVEF is key to predicting worse clinical outcomes, particularly in patients with several conventional risk factors such as high age, male sex, heart failure, and renal impairment. 

Notably, the persistence of extra-valvular abnormalities even after successful aortic valve correction through TAVR has been recognized. Consequently, a significant proportion of TAVR candidates may be characterized as having HFpEF following the alleviation of aortic valve stenosis. Therefore, the pathophysiological mechanisms at play in our patient cohorts may closely mirror those observed in individuals with HF and snLVEF, further emphasizing the clinical significance of snLVEF in the context of TAVR. 

### 4.4. Estimated Underlying Mechanism of Our Findings

The precise underlying mechanisms driving these findings remain to be fully elucidated. However, previous research has offered some potential explanations and avenues for further exploration. In a prior study, it was observed that women with snLVEF faced an elevated risk of cardiac events, possibly linked to heightened microvascular dysfunction and an increase in sympathetic tone [16]. These factors may contribute to the adverse outcomes associated with snLVEF. 

Another study, involving individuals undergoing coronary computed tomography angiography, found that snLVEF was associated with worse outcomes in women, who typically have smaller hearts compared to men [17]. This observation raises the possibility that individuals with smaller hearts may require a higher ejection fraction to maintain sufficient cardiac output. This increased demand on the heart’s pumping function could lead to greater oxygen consumption [18], potentially contributing to the incremental adverse outcomes observed in individuals with snLVEF. 

An alternative explanation for our findings could involve the presence of sub-clinical diseases that lead to an increase in LVEF while concurrently exacerbating clinical outcomes [19]. These sub-clinical conditions might encompass a range of health issues, such as anemia, hyperthyroidism, systemic infection, obesity, and arteriovenous shunts. These conditions could potentially elevate LVEF levels but also contribute to worse clinical outcomes. It is worth noting that, despite the presence of multiple comorbidities among the TAVR candidates in our study, many of these conditions were relatively well controlled before the TAVR procedure. This suggests that while these sub-clinical diseases may have contributed to the observed outcomes in some cases, they may not have been the primary drivers of the associations between snLVEF and adverse clinical outcomes. 

Following TAVR, patients with snLVEF had relatively higher LVOT peak velocity than their counterparts. Unmasked LV dynamic obstruction due to hyper-construction may cause deteriorated hemodynamics and worse clinical outcomes in patients with snLVEF [20]. However, only a few patients had significant LVOT obstruction. This finding may not explain the whole underlying mechanism. 

In addition to environmental factors, the genetic disorder may also be a key to explaining the pathophysiology of snLVEF. In a recent study, genetic predisposition was associated with the presence of underdiagnosed HF and higher mortality among population-based cohorts with snLVEF [21]. Further studies are warranted to clarify the physiological mechanism of snLVEF and to construct a therapeutic strategy for the TAVR candidates with snLVEF.

We investigated the detailed profiles and prognostic impact of snLVEF in the TAVR candidates for the first time using a large-scale multi-institutional registry with comprehensive data. This study should be a proof-of-concept for further studies involving snLVEF in patients with a variety of diseases. 

### 4.5. Limitations

It is important to recognize the specific characteristics of the OCEAN-TAVI registry, which primarily comprises Japanese patients with severe aortic stenosis who often present with high-risk comorbidities, particularly older age. Consequently, the generalizability of our findings to other patient cohorts remains uncertain. The unique patient population within the registry may limit the direct applicability of our results to broader populations with different demographic and clinical profiles.

The inherent limitations of utilizing a large-scale, multi-institutional registry database should be acknowledged. Such databases may lack detailed clinical data, including invasive hemodynamics information, which could provide additional insights into the underlying mechanisms and intricacies of our findings. We could not perform a comprehensive screening of cardiac amyloidosis, which may have been one of the potential confounders that have a negative prognostic impact [22]. 

Our study assessed echocardiographic data at a single time point before TAVR, and we do not possess data regarding the trends in snLVEF over the extended observation period following TAVR [23]. Consequently, we are unable to elucidate how snLVEF may evolve and its potential prognostic implications beyond the initial assessment.

Contrary to the substantial prognostic influence observed for snLVEF within the multivariable analysis, the presence of snLVEF did not yield a statistically significant effect on the primary outcome within the univariable analysis. Patients exhibiting snLVEF displayed relatively younger age, less male sex, conserved renal function, and presented with less advanced heart failure at baseline, all of which had a negative (favorable) prognostic impact in the multivariable analysis. These plausible confounding variables may have obscured its prognostic impact within the univariable analysis. Consistently, patients who exhibited both snLVEF and at least one of these risk factors were at higher risk of the primary outcome than their counterparts. 

## 5. Conclusions

The prevalence of snLVEF is notably common among TAVR candidates. Our findings suggest that patients with snLVEF may exhibit a distinct clinical phenotype and experience worse clinical outcomes following TAVR in comparison to those with nLVEF. These observations underscore the need for further in-depth studies to elucidate the underlying physiological mechanisms driving snLVEF and to develop tailored therapeutic strategies for TAVR candidates with this specific condition.

As we strive to improve patient care and outcomes in the context of TAVR, it is imperative to gain a deeper understanding of snLVEF and its implications. This will not only help refine risk stratification for TAVR candidates but also inform the development of more effective treatment approaches to optimize outcomes for this unique patient population. Further research endeavors should aim to unravel the intricacies of snLVEF and contribute to the advancement of clinical care in the field of TAVR.

## Figures and Tables

**Table 1:** Baseline characteristics.

	Total (*N* = 5989)	snLVEF (*N* = 2819)	nLVEF (*N* = 3170)	*p*-Value
Demographics				
Age, years	84.4 ± 5.1	84.2 ± 5.1	84.6 ± 5.1	0.002 *
Male sex	1783 (30%)	742 (26%)	1041 (33%)	<0.001 *
Body mass index	22.5 ± 3.8	22.6 ± 3.8	22.5 ± 3.7	0.24
New York Heart Association class III/IV	2187 (37%)	891 (32%)	1296 (41%)	<0.001 *
Comorbidity				
Hypertension	5063 (85%)	2343 (83%)	2720 (86%)	0.002 *
Diabetes mellitus	1557 (26%)	709 (25%)	848 (27%)	0.085
Dyslipidemia	3339 (56%)	1594 (57%)	1745 (55%)	0.13
Atrial fibrillation	1202 (20%)	501 (18%)	701 (22%)	<0.001 *
Chronic kidney disease	4078 (68%)	1859 (66%)	2219 (70%)	<0.001 *
History of stroke	667 (11%)	326 (12%)	341 (11%)	0.17
Peripheral artery disease	644 (11%)	288 (10%)	356 (11%)	0.11
Chronic obstructive pulmonary disease	569 (10%)	245 (9%)	324 (10%)	0.024 *
History of percutaneous coronary intervention	1267 (21%)	531 (19%)	736 (23%)	<0.001 *
History of coronary artery bypass graft	213 (4%)	83 (3%)	130 (4%)	0.009 *
History of myocardial infarction	192 (3%)	49 (2%)	143 (5%)	<0.001 *
Laboratory data				
Hemoglobin, g/dL	11.4 ± 1.6	11.4 ± 1.6	11.4 ± 1.7	0.70
Serum albumin, g/dL	3.8 ± 0.5	3.8 ± 0.5	3.7 ± 0.5	<0.001 *
Serum sodium, mEq/L	140.0 ± 3.3	140.0 ± 3.4	140.1 ± 3.3	0.26
eGFR, mL/min/1.73m^2^	52.1 ± 18.9	53.1 ± 18.7	51.1 ± 19.0	<0.001 *
Logarithm of plasma BNP, pg/mL	2.28 ± 0.46	2.20 ± 0.45	2.37 ± 0.45	<0.001 *
Echocardiographic data				
LVDd, mm	42.7 ± 5.7	41.8 ± 5.4	43.5 ± 5.8	<0.001 *
LVEF, %	64.8 ± 7.3	71.0 ± 4.5	59.4 ± 4.2	<0.001 *
Left atrial diameter, mm	41.8 ± 7.3	41.3 ± 7.4	42.2 ± 7.2	<0.001 *
Interventricular septum diameter, mm	11.9 ± 2.2	11.9 ± 2.2	11.9 ± 2.1	0.96
Posterior wall diameter, mm	11.3 ± 2.0	11.3 ± 2.0	11.4 ± 2.0	0.64
Moderate or greater AR	569 (10%)	235 (8%)	334 (11%)	0.002 *
Moderate or greater MR	543 (9%)	236 (8%)	307 (10%)	0.043 *
Moderate or greater TR	494 (8%)	238 (8%)	256 (8%)	0.32
Peak velocity at aortic valve, m/s	4.59 ± 0.77	4.62 ± 0.75	4.57 ± 0.79	0.10
Mean pressure gradient at aortic valve, mmHg	50.6 ± 18.2	50.6 ± 17.8	50.5 ± 18.6	0.82
STS score	7.1 ± 4.8	6.7 ± 4.5	7.4 ± 5.0	<0.001 *

snLVEF, supra-normal left ventricular ejection fraction; nLVEF, normal left ventricular ejection fraction; eGFR, estimated glomerular filtration rate; BNP, B-type natriuretic peptide; LVDd, left ventricular end-diastolic diameter; LVEF, left ventricular ejection fraction; AR, aortic regurgitation; MR, mitral regurgitation; TR, tricuspid regurgitation; STS, society of thoracic surgeon. Continuous variables were presented as mean and standard deviation and compared between the two groups via unpaired *t*-test or Mann–Whitney U test according to their distribution. Categorical variables were presented as numbers and percentages and compared between the two groups using Chi-square test or Fischer’s exact test as appropriate. * *p* < 0.05. All participants had LVEF ≥ 50%. snLVEF was defined as LVEF > 65% and nLVEF as 50% ≤ LVEF ≤ 65%.

**Table 2:** Post-procedural adverse events.

	snLVEF (*N* = 2819)	nLVEF (*N* = 3170)	*p*-Value
Peri-procedural complication			
Acute kidney injury	209 (7%)	253 (8%)	0.22
Cardiac tamponade	30 (1%)	28 (0.9%)	0.28
Valve embolization	11 (0.4%)	5 (0.2%)	0.068
In-hospital complication			
Coronary obstruction	12 (0.4%)	24 (0.8%)	0.067
Ischemic stroke	54 (2%)	76 (2%)	0.12
Hemorrhagic stroke	5 (0.2%)	6 (0.2%)	0.58
Disabling stroke	23 (0.8%)	35 (1%)	0.16
Transient ischemic attack	3 (0.1%)	6 (0.2%)	0.32
Major bleeding	201 (7%)	199 (6%)	0.10
Pacemaker implantation	227/2691 (8%)	261/3006 (9%)	0.39
New atrial fibrillation	63 (2%)	97 (3%)	0.029 *
Major vascular complication	102 (4%)	103 (3%)	0.24

snLVEF, supra-normal left ventricular ejection fraction; nLVEF, normal left ventricular ejection fraction. Categorical variables were presented as numbers and percentages and compared between the two groups using Chi-square test or Fischer’s exact test as appropriate. * *p* < 0.05. All participants had LVEF ≥ 50%. snLVEF was defined as LVEF > 65% and nLVEF as 50% ≤ LVEF ≤ 65%.

**Table 3:** One-year echocardiography follow-up.

	snLVEF (*N* = 405)	nLVEF (*N* = 252)	*p* Value
LVDd, mm	41.6 ± 5.0	43.1 ± 5.3	<0.001 *
LVEF, %	71.8 ± 7.2	67.5 ± 9.8	<0.001 *
Interventricular septum diameter, mm	10.3 ± 2.3	10.2 ± 2.1	0.47
Posterior wall diameter, mm	9.8 ± 2.0	9.8 ± 1.7	0.79
Moderate or greater AR	17 (4%)	18 (7%)	0.074
Moderate or greater MR	30 (7%)	26 (10%)	0.12
Moderate or greater TR	32 (8%)	28 (11%)	0.11
Peak velocity at aortic valve, m/s	2.40 ± 0.51	2.23 ± 0.44	<0.001 *
Mean pressure gradient at aortic valve, mmHg	12.6 ± 5.6	10.8 ± 4.7	<0.001 *
LVOT peak velocity, m/s	1.07 ± 0.32	0.98 ± 0.23	0.017 *
LVOT peak velocity > 2.0 m/s	9 (2%)	0 (0%)	0.017 *

snLVEF, supra-normal left ventricular ejection fraction; nLVEF, normal left ventricular ejection fraction; LVDd, left ventricular end-diastolic diameter; LVEF, left ventricular ejection fraction; AR, aortic regurgitation; MR, mitral regurgitation; TR, tricuspid regurgitation; LVOT, left ventricular outflow tract. Continuous variables were presented as mean and standard deviation and compared between the two groups via unpaired *t*-test or Mann–Whitney U test according to their distribution. Categorical variables were presented as numbers and percentages and compared between the two groups using Chi-square test or Fischer’s exact test as appropriate. * *p* < 0.05. All participants had LVEF ≥ 50%. snLVEF was defined as LVEF > 65% and nLVEF as 50% ≤ LVEF ≤ 65%.

**Table 4:** Prognostic impact of the presence of snLVEF on three-year clinical outcome after TAVR.

	Unadjusted Analyses		Adjusted Analyses	
	Hazard Ratio (95% CI)	*p*-Value	Hazard Ratio (95% CI)	*p*-Value
Death or heart failure readmission				
snLVEF versus nLVEF	0.93 (0.83–1.03)	0.16	1.16 (1.02–1.31)	0.023 *
LVEF, %	0.99 (0.98–1.00)	0.047 *	1.01 (1.00–1.02)	0.030 *
Death				
snLVEF versus nLVEF	0.91 (0.81–1.04)	0.16	1.20 (1.04–1.38)	0.014 *
LVEF, %	0.99 (0.98–1.00)	0.021 *	1.01 (0.99–1.02)	0.055
Heart failure readmission				
snLVEF versus nLVEF	0.87 (0.72–1.04)	0.12	0.95 (0.77–1.18)	0.64
LVEF, %	0.99 (0.98–1.01)	0.091	1.01 (0.99–1.02)	0.097

TAVR, trans-catheter aortic valve replacement; snLVEF, supra-normal left ventricular ejection fraction; nLVEF, normal left ventricular ejection fraction; LVEF, left ventricular ejection fraction; CI, confidence interval. Cox proportional hazard ratio regression analyses were performed for snLVEF as a dichotomized variable or LVEF as a continuous variable to predict 3-year clinical outcomes. Hazard ratios were adjusted for pre-specified potential confounders including age, male sex, the presence of chronic kidney disease, and the logarithm of plasma B-type natriuretic peptide level. * *p* < 0.05. All participants had LVEF ≥ 50%. snLVEF was defined as LVEF > 65% and nLVEF as 50% ≤ LVEF ≤ 65%.

## Data Availability

Data are available from the corresponding author upon the reasonable request.
